# Diagnostic and therapeutic potential of tonic gamma‐aminobutyric acid from reactive astrocytes in brain diseases

**DOI:** 10.1002/ctm2.1642

**Published:** 2024-04-01

**Authors:** Wuhyun Koh, C. Justin Lee

**Affiliations:** ^1^ Center for Cognition and Sociality Life Science Cluster Institute for Basic Science (IBS) Daejeon South Korea

**Keywords:** astrocyte, MAO‐B, putrescine, tonic GABA

Dear Editor,

Gamma‐aminobutyric acid (GABA), the primary inhibitory neurotransmitter in the brain, serves as a cornerstone in modulating neuronal activity and maintaining cognitive functions. Its role extends beyond fast‐acting phasic inhibition, as slow‐acting tonic GABA inhibition, mediated by extrasynaptic GABA_A_ receptors, responds to ambient GABA levels (i.e. GABA tone), significantly influencing synaptic plasticity, learning and memory, and sensory processing.^1^ Dysregulation of tonic GABA inhibition in a range of neurological and psychiatric diseases is associated with cognitive decline, motor symptoms, and other functional impairments, highlighting the potentially broad impact of GABA tone across various diseases. One of the common cellular responses in pathological conditions is the emergence of reactive astrocytes,^2^ often accompanied by upregulation of monoamine oxidase B (MAO‐B) expression and increased GABA synthesis (Figure 1A), thus affecting neighbouring neurons through tonic inhibition.^3^ Here, we present the impact of pathological GABA tone and propose its therapeutic and diagnostic targets for clinical applications.

**FIGURE 1:**
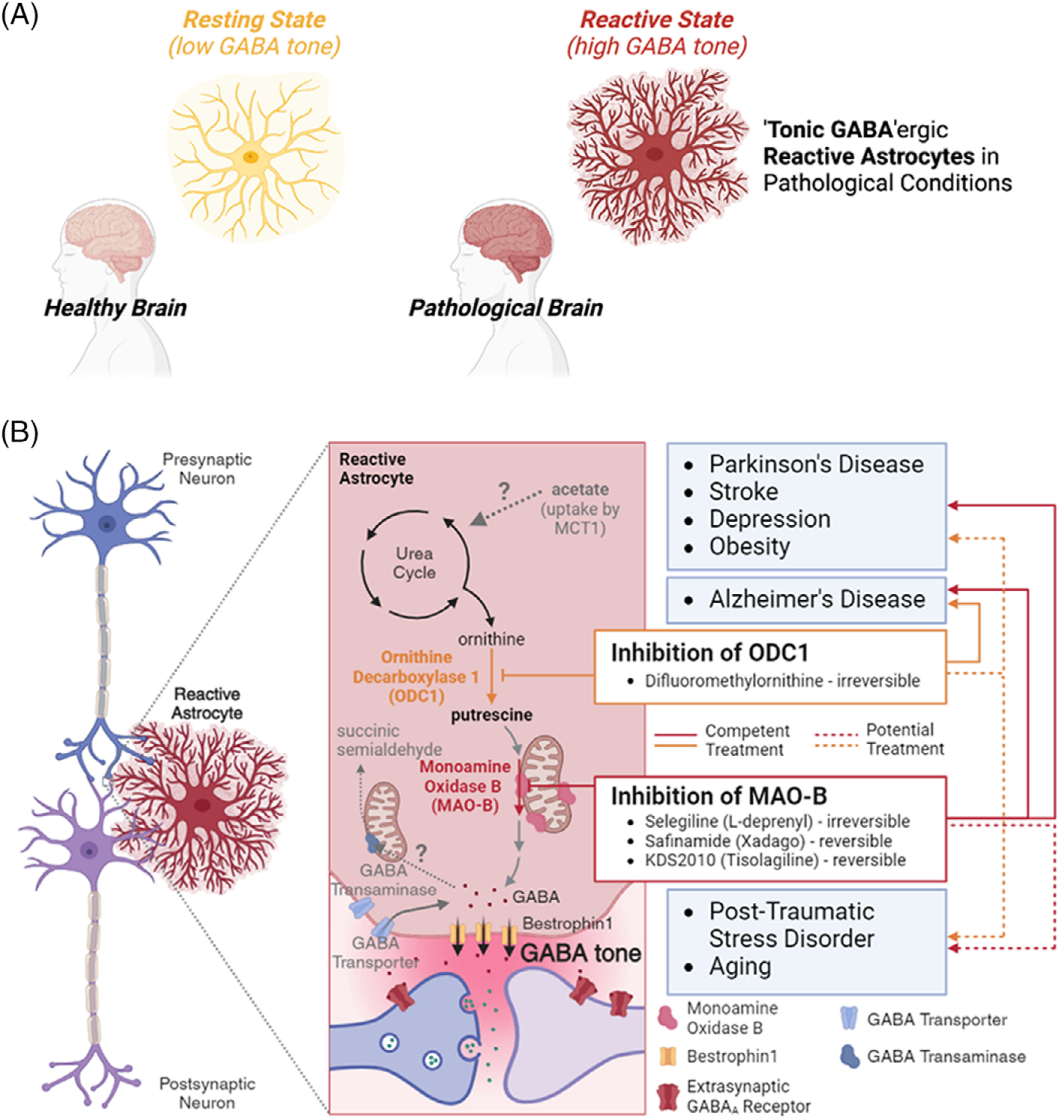
'Tonic GABA'ergic reactive astrocyte and gamma‐aminobutyric acid (GABA) synthesis mechanisms from astrocytes in pathological brain and related brain diseases with therapeutic targets. (A) In the healthy brain, astrocytes are in a resting state, whereas in the pathological brain, astrocytes change to a reactive state with increased monoamine oxidase B (MAO‐B) expression and GABA synthesis. As a result, GABA release from astrocytes increases and leads to an increase in GABA tone in pathological brain regions. (B) The reactive astrocyte increases GABA tone in the pathologic region, leading to the development of pathologic symptoms. The reactive astrocyte primarily utilizes MAO‐B enzymes in the mitochondria to degrade putrescine to synthesize GABA. Therefore, inhibition of MAO‐B can be a competent treatment for a variety of diseases that can be mediated by reactive astrocytes (Parkinson's disease, stroke, depression, obesity and Alzheimer's disease), and may also be a potential treatment for posttraumatic stress disorder and ageing. In addition, ornithine decarboxylase 1 (ODC1), which can make putrescine, a precursor for GABA, from ornithine in the urea cycle, has been shown to be a competent treatment for Alzheimer's disease and may be a potential treatment for other MAO‐B‐related brain disorders in general.

Astrocytes once thought to be mere support cells, emerge as key players in the regulation of GABA tone through various mechanisms of GABA synthesis, release, and clearance.^1^ These cells are strategically positioned to interface closely with neurons, allowing them to effectively regulate GABA tone in the extrasynaptic space. This role becomes particularly crucial in a range of pathological conditions, such as Alzheimer's^3^, ^4^, ^5^ and Parkinson's diseases.^6^, ^7^ In these conditions, astrocytes become reactive, exhibiting increased MAO‐B expression and enhanced GABA synthesis, which contributes to an elevated GABA tone and leads to pathological symptoms. This phenomenon is not limited to Alzheimer's and Parkinson's disease but is also observed in stroke,^8^ depression,^9^ obesity,^10^ and potentially extends to conditions like post‐traumatic stress disorder (PTSD)^11^ and ageing.^12^ In Alzheimer's disease, reactive astrocytes in the hippocampus of APP/PS1 mouse models and the temporal cortex of human postmortem brains exhibit increased levels of MAO‐B and GABA.^3^ This elevation in astrocytic GABA leads to enhanced tonic inhibition, reducing spike probability and long‐term potentiation in neurons, thereby impairing learning and memory.^3^ Similarly, in Parkinson's disease, augmented GABA levels in reactive astrocytes increase tonic inhibition in the substantia nigra pars compacta (SNpc) in A53T, MPTP mouse models, and 6‐OHDA rat model, resulting in parkinsonian motor symptoms.^7^ It is noteworthy that this tonic inhibition reduces not only the activity of SNpc cells but also the tyrosine hydroxylase (TH) expression of SNpc neurons, significantly impacting their function without extensive death. Clinically relevant, in Parkinson's disease patients, despite a marked reduction in TH expression in SNpc neurons, a substantial number of these neurons remain viable in the mild stages, contrasting with their extensive death in the severe stages.^7^ In stroke, diaschisis, characterized by glucose hypometabolism in regions distant from the injury site, is exacerbated by elevated GABA tone via reactive astrocytes.^8^ This elevated GABA tone inhibits synaptic activity in adjacent cortical neurons, hindering functional recovery, particularly in motor deficits.^8^ In depression, increased tonic inhibition due to MAO‐B‐dependent GABA synthesis has been reported in the hippocampus of social deprivation mice^13^ and the medial prefrontal cortex of Flinders Sensitive Line rat models^9^ of depression, leading to suppressed long‐term potentiation. A similar mechanism is suspected in PTSD, where increased prefrontal GABA^11^ might originate from reactive astrocytes, this hypothesis awaiting further investigation. In obesity, reactive astrocytes induced by a high‐fat diet have been found to synthesize and release GABA in the lateral hypothalamic area, inhibiting GABRA5‐expressing neurons and contributing to weight gain.^10^ Additionally, the increase in MAO‐B during aging^12^ suggests that augmented GABA from reactive astrocytes may contribute to cognitive decline, meriting further research. Collectively, these findings underscore the significant impact of MAO‐B‐dependent GABA synthesis in reactive astrocytes across brain diseases, highlighting the promise of targeting GABA tone reduction through MAO‐B inhibition for clinical applications.

Despite the promise of MAO‐B inhibitors in treating brain diseases, a limitation of drugs like selegiline (L‐deprenyl) is their effects are not long‐lasting. In APP/PS1 mice, selegiline showed a therapeutic effect lasting approximately one week, but this effect diminished with long‐term administration of about four weeks.^3^, ^5^ Notably, prolonged use of selegiline triggered a compensatory mechanism involving diamine oxidase (DAO)‐dependent GABA synthesis, a pathway alternative to MAO‐B that degrades putrescine into GABA.^5^ As an irreversible MAO‐B inhibitor, selegiline forms a covalent bond with MAO‐B, eventually destroying it and subsequently activating the compensatory mechanism (i.e. DAO‐dependent GABA synthesis). On the other hand, reversible MAO‐B inhibitors such as safinamide (Xadago) and the newly developed KDS2010 (Tisolagiline) have less compensatory effects because they compete with the substrate and consequently leave MAO‐B intact.^5^ This contrast strongly suggests the use of reversible, but not irreversible, MAO‐B inhibitors as a long‐term treatment to reduce MAO‐B‐dependent GABA synthesis in pathological conditions. Among the reversible MAO‐B inhibitors under active investigation, safinamide, which is approved by the US Food and Drug Administration for Parkinson's disease, also inhibits voltage‐dependent Na^+^ and Ca^2+^ channels. Meanwhile, the recently developed KDS2010, having successfully completed Phase I clinical trial, is expected to enter Phase II clinical trial for Alzheimer's disease and obesity in 2024. Auspiciously, in animal models, KDS2010 is effective not only for Alzheimer's disease^5^ but also for Parkinson's disease,^6^ obesity,^10^ diaschisis in stroke recovery.^8^ KDS2010 may also be applicable in depression, where inhibition of MAO‐B restores the impairments,^9^, ^13^ and in ageing, where MAO‐B is upregulated.^12^ Thus, reversible MAO‐B inhibitors hold promise as therapeutic drugs for a range of brain diseases linked to MAO‐B‐dependent GABA synthesis and release from reactive astrocytes.

Inhibiting MAO‐B and related metabolic pathways offers several benefits beyond merely reducing GABA tone in treating various diseases. First, the inhibition of MAO‐B is generally safe, as evidenced by the minimal effects in cases of MAO‐B deficiency.^14^ Additionally, inhibiting MAO‐B can also suppress the generation of hydrogen peroxide (H_2_O_2_), a byproduct in MAO‐B‐dependent GABA synthesis, which in turn can induce astrocytic inducible nitric oxide synthase (iNOS) expression, nitrosative stress, and microglial activation.^15^ Notably, these symptoms were mitigated by the use of KDS2010 or a potent H_2_O_2_ scavenger, suggesting that MAO‐B‐dependent H_2_O_2_ production intensifies astrocyte reactivity and pathological symptoms. This implies that an effective H_2_O_2_ scavenging drug could also be a promising clinical approach for diseases featured by MAO‐B‐dependent GABA tone increase from reactive astrocytes. Furthermore, inhibition of ornithine decarboxylase 1 (ODC1), which is upstream of MAO‐B in the putrescine degradation pathway from the urea cycle, not only reduces the synthesis of GABA and H_2_O_2_ but also helps in eliminating toxic molecules like β‐amyloid, relieving symptoms of Alzheimer's disease.^4^, ^16^ In addition, exploring the modulation of GABA tone via GABA transaminase (also known as 4‐aminobutyrate aminotransferase), which plays a key role in the degradation of intracellular GABA, is crucial and could provide significant therapeutic targets. Taken together, these findings suggest that targeting GABA synthesis via MAO‐B inhibition could be a promising therapeutic approach for related brain diseases (Figure 1B).

Finally, the elevated GABA tone resulting from increased MAO‐B activity in reactive astrocytes can serve as a potential diagnostic marker for brain diseases. Neuroimaging of ^1^H‐ magnetic resonance spectroscopy (MRS) GABA signal has been suggested to reflect tonic rather than phasic inhibition in the brain,^17^ indicating that variations in GABA tone, particularly from increased MAO‐B activity in reactive astrocytes, can be instrumental in diagnosing various brain diseases. Moreover, the advancement in positron emission tomography (PET) imaging, with agents targeting reactive astrocytes^18^ or MAO‐B^19^ such as [^11^C]‐L‐deprenyl, [^11^C]‐L‐deprenyl D2, [^11^C]SL25.1188, [^18^F]THK‐5351, [^18^F]‐fluorodeprenyl‐D2, [^18^F]‐SMBT‐1, has opened new possibilities in identifying pathological brain regions in patients. Recently, PET imaging with ^11^C‐acetate and ^18^F‐fluorodeoxyglucose (FDG) visualized reactive astrocyte‐associated neuronal glucose hypometabolism for AD patients, and discovered a strong correlation between the patient's cognitive function and the PET signals of both ^11^C‐acetate and ^18^F‐FDG,^20^ demonstrating the potential value of PET imaging by visualizing reactive astrogliosis and the associated neuronal glucose hypometabolism for AD and brain diseases. Note that the strong correlation between reactive astrocyte‐mediated acetate metabolism and neuronal hypometabolism with the cognitive decline in Alzheimer's Disease patients suggests that acetate's involvement in GABA metabolism in reactive astrocytes warrants further study. In summary, these developments underscore the potential of GABA tone, alongside markers of reactive astrocytes, as effective diagnostic biomarkers for a broad spectrum of brain diseases, emphasizing the need for further identification of markers to enhance comprehensive diagnostic and treatment strategies.

In conclusion, the regulation of GABA tone, especially through astrocytes, plays a crucial role in our understanding of both brain function and dysfunction. The observation of increased GABA tone alongside reactive astrocytes in various brain diseases highlights the urgent need for foundational research to explore their underlying mechanisms. Additionally, clinical studies are necessary to determine their potential effects. This two‐pronged approach is vital for a thorough comprehension of the pathological changes and the development of effective treatments for these diseases. Understanding GABA tone is key to discovering therapeutic interventions and developing biomarkers for numerous neurological conditions. This expanding field of research holds great promise for advancing our knowledge and treatment of a wide range of brain diseases.

## AUTHOR CONTRIBUTIONS

Not applicable.

## CONFLICT OF INTEREST STATEMENT

The authors declare no conflict of interest.

## FUNDING INFORMATION

Center for Cognition and Sociality, Gran/Award Number: IBS‐R001‐D2 to C. Justin Lee; Young Scientist Fellowship Gran/Award Number: IBS‐R001‐Y1 to Wuhyun Koh.

## ETHICS STATEMENT

Not applicable.
